# A multivariate approach to the integration of multi-omics datasets

**DOI:** 10.1186/1471-2105-15-162

**Published:** 2014-05-29

**Authors:** Chen Meng, Bernhard Kuster, Aedín C Culhane, Amin Moghaddas Gholami

**Affiliations:** 1 Chair of Proteomics and Bioanalytics, Technische Universität München, Freising, Germany; 2 Center for Integrated Protein Science Munich, Freising, Germany; 3 Department of Biostatistics and Computational Biology, Dana-Farber Cancer Institute, Boston, MA 02215, USA; 4 Department of Biostatistics, Harvard School of Public Health, Boston, MA 02215, USA

**Keywords:** Multivariate analysis, Multiple co-inertia, Data integration, Omic data, Visualization

## Abstract

**Background:**

To leverage the potential of multi-omics studies, exploratory data analysis methods that provide systematic integration and comparison of multiple layers of omics information are required. We describe multiple co-inertia analysis (MCIA), an exploratory data analysis method that identifies co-relationships between multiple high dimensional datasets. Based on a covariance optimization criterion, MCIA simultaneously projects several datasets into the same dimensional space, transforming diverse sets of features onto the same scale, to extract the most variant from each dataset and facilitate biological interpretation and pathway analysis.

**Results:**

We demonstrate integration of multiple layers of information using MCIA, applied to two typical “omics” research scenarios. The integration of transcriptome and proteome profiles of cells in the NCI-60 cancer cell line panel revealed distinct, complementary features, which together increased the coverage and power of pathway analysis. Our analysis highlighted the importance of the leukemia extravasation signaling pathway in leukemia that was not highly ranked in the analysis of any individual dataset. Secondly, we compared transcriptome profiles of high grade serous ovarian tumors that were obtained, on two different microarray platforms and next generation RNA-sequencing, to identify the most informative platform and extract robust biomarkers of molecular subtypes. We discovered that the variance of RNA-sequencing data processed using RPKM had greater variance than that with MapSplice and RSEM. We provided novel markers highly associated to tumor molecular subtype combined from four data platforms. MCIA is implemented and available in the R/Bioconductor “omicade4” package.

**Conclusion:**

We believe MCIA is an attractive method for data integration and visualization of several datasets of multi-omics features observed on the same set of individuals. The method is not dependent on feature annotation, and thus it can extract important features even when there are not present across all datasets. MCIA provides simple graphical representations for the identification of relationships between large datasets.

## Background

There has been rapid progress in high-throughput technologies and platforms to assay global mRNA, miRNA, methylation, proteins, and metabolite profiles of cells are readily available. Advances in RNA-sequencing and mass spectrometry (MS) based proteomics have dramatically improved coverage and quality of genomic, transcriptomic and proteomic profiling [1-4]. Increasing number of studies including The Cancer Genome Atlas (TCGA) and ENCyclopedia of DNA Elements (ENCODE) projects systematically profile large number of biological samples resulting in multiple levels of quantitative information [5-8]. Recent advances of MS based proteomics provide a complementary approach to genomics and transcriptomic technologies [3,4] and systematic analyses can now be carried out to identify and quantify the majority of proteins expressed in human cells [9-12]. These data yield unprecedented views of molecular building blocks and the machinery of cells. Interpreting these large-scale datasets to derive information about a biological system represents a considerable challenge often faced by investigators.

Multiple omics data analysis can be broadly defined by some common questions, which are dependent on the data collected; multiple datasets measuring the same biological molecules or multiple datasets each measuring different biological molecules. In the first case, given multiple transcriptomics data from different microarray or RNA-sequencing studies, the aim may be to discover which platform is the most informative with highest quality data, identify robust biomarkers across datasets or highlight platform specific discrepancies in measurements. In the second case, given multiple different data such as transcripts, proteins and metabolites, the objective may be to integrate and concatenate information to increase the breath and coverage of available data in a biological network. In this case, specific platform discrepancies are less important and performance of data integration is more likely to be assessed using system biology or pathway approaches.

Nevertheless, both analyses face common challenges associated with integrating data from disparate technologies. Several meta-analysis studies map identifiers from each platform to a common set of identifiers to generate a single concatenated matrix for subsequent analysis [13,14]. However, this data simplification overlooks several fundamental platform and biological biases. Platforms are not universal and measure different molecules. Filtering genes to their intersection may considerably reduce data coverage. In addition, the many-to-many mapping of gene identifiers from multiple platforms complicates direct comparison of molecules across multiple levels. Moreover, because correlations between different platforms are probably lower than expected [15], it may not provide gains in data quality or study power. Such filtering may also introduce bias because platform discrepancies could reflect biological variation. For instance, poor correlation between a transcript and its translated protein may result from biological processes such as microRNA post-transcriptional repression [16,17]. Similarly, correlations between proteins and metabolites of pathways can diverge if proteins are expressed in an inactive form, in which case its abundance may not represent activity.

Ordination methods, such as principal component analysis (PCA), independent component analysis (ICA) and correspondence analysis (COA), are exploratory data analysis approaches that have been applied to analyze omics data including transcriptome and proteome studies [18-22]. Graphical representation of measurements (samples) and variables (genes, proteins) on a lower dimensional space facilitates interpretation of global variance structure and identification of the most informative (or variant) features across datasets. These methods permit visualization of data that have considerable levels of noise and data where the number of variables exceeds the number of measurements, which is typical in omics studies. However, these approaches do not solve the problem of comparing many datasets simultaneously.

Studies have extended these approaches to couple two datasets together [23]. One such approach is co-inertia analysis (CIA) [24]. CIA was originally applied to study ecological and environmental tables, where it was employed to link environmental variables with species characteristics [25]. Culhane and colleagues introduced CIA in genomics, when they compared data from two microarray platforms [26]. An advantage of this method is that it does not require the mapping or filtering of genes to a common set. The relationship between co-inertia analysis and related methods including Procrustes analysis [24], canonical correlation analysis with Elastic Net penalization (CCA-EN) and sparse Partial Least Squares (sPLS) have been described previously [27]. CIA and sPLS both maximize the covariance between eigenvectors and are efficient in determining main individual effects in paired dataset analysis. By contrast CCA-EN maximizes the correlation between eigenvectors and tends to discover effects present in both datasets, but may omit to discover strong individual effects. Variables selected by CCA-EN and sPLS are highly similar but CIA selected marginally different marker genes that may have some redundancy [27]. A noteworthy advantage of CIA is that it can be coupled with several dimension reduction approaches, including PCA or correspondence analysis, such that it can accommodate both discrete count data (e.g. somatic mutation) and continuous data [26]. These approaches are performed on each dataset separately and can be integrated using CIA [24]. However, all above methods including CIA are limited to the analysis of two datasets, limiting their application in modern multi-omics studies. Several approaches have been proposed for integrating more than two datasets, such as consensus PCA (CPCA) [28], regularized generalized canonical correlation analysis (RGCCA) [29], sparse generalized canonical correlation analysis (SGCCA) [30] and penalized canonical correlation analysis (PCCA) [31]. SGCCA and PCCA originally focus on the feature selection from multiple datasets, but also can be used for multiple table integration problem.

Here, we describe another method, multiple co-inertia analysis (MCIA), for the analysis of more than two omics datasets, extending its application in the field of environmental science and, recently, phylogenetics [32]. MCIA is related to consensus PCA (CPCA) which both maximize the square covariance between eigenvectors and are subject to similar constraints [28]. CPCA is less sensitive to multi-collinearity within each dataset than generalized canonical correlation analysis [28]. We illustrate the application of MCIA using two different examples, and show that integrated analysis is more insightful than analysis of the individual datasets. First, we demonstrated the power of MCIA via applying it to the integration and comparison of multi-omics data independent of data annotation. We employed MCIA to identify common relationships among multiple gene and protein expression data of the NCI-60 cancer cell line panel of the National Cancer Institute [8,11,33]. The integrated analysis revealed that cell lines are clustered according to anatomical tissue source and showed a significant degree of correlation between transcript and protein expression. Second, we assessed the concordance in gene expression data obtained from microarray and next generation RNA-sequencing of 266 samples of high grade serous ovarian cancer. MCIA integrated ovarian cancer gene expression data from different sources which captured distinct subsets of the transcriptome (<47% of genes were present on all four platforms) to reveal a set of biomarkers that were consistently highly ranked by all four platforms and were biologically relevant to ovarian cancer. To enable community access to MCIA, we implemented the method into the R-Bioconductor (omicade4) package as an easy-to-use tool for bioinformaticians and biologists.

## Methods

### Mathematical basis of MCIA

A typical omics dataset is a matrix where the number of features exceeds the number of measurements (rows and columns of the matrix, respectively). MCIA requires a set of tables where either features or measurements are matched and have equal weights. MCIA is performed in a two-step process. First a one table ordination method, such as PCA, COA or non-symmetric correspondence analysis (NSC) [34] is applied on each dataset separately, which transforms data into comparable lower dimensional spaces.

In our analysis, given an omics data table **M** = [m_ij_] with 1 ≤ i ≤ n and 1 ≤ j ≤ q, where **M** is a (*n* x *q)* matrix, *i* indicates row index and *j* for column index. We denote the row and column sums of **M** as m_i+_ and m_+j_ respectively, and m_++_ as the grand total. The relative contribution or weight of row *i* to the total variation in the data set is denoted r_i_ and calculated as r_i_ = m_i+_/m_++_ while the relative contribution of column *j* is denoted as c_j_ = m_+j_/m_++_. Similarly, the contribution of each individual element of **M** to the total variation p_ij_ can be calculated as p_ij_ = m_ij_/m_++_. We then derive a new matrix **X** with the values defined above as

(1) $$x_{\mathrm{ij}}=\frac{p_{\mathrm{ij}}}{r_{i}}-c_{j}$$

where x_ij_ is the centered row profile, i.e. the relative abundance of selected variable to the measurement’s weight.

The second step in MCIA is a generalization of CIA [26]. It solves the problem of simultaneous analysis of a set of statistical triplet (**X**_**k**_, **Q**_**k**_, **D**) where k = 1, …, k,…, K and **X**_**k**_ is a set of transformed matrices. **Q**_**k**_ is a q_k_ × q_k_ matrix with r_ij_ in diagonal elements, indicating the hyperspace of features metrics. **D** is an n × n matrix which is an identity matrix indicating equal weight across all columns in all tables. MCIA maximizes the sum of the squared covariance between scores of each table with synthetic axes ν, that is:

(2) $$f\left(u_{1},\ldots ,u_{k},\ldots ,u_{K},v\right)=\sum_{k=1}^{K}w_{k}\mathrm{co}v^{2}\left(X_{k}Q_{k}u_{k},v\right)$$

where *cov*^*2*^ stands for the square of covariance of quantities inside parenthesis and ω_*k*_ is the weight of each table. The **v** represents the reference structure or synthetic center and **u**_**k**_ are auxiliary axes. The score of each individual table would then be **X**_**k**_**Q**_**k**_**u**_**k**_. In contrast with other ordination methods, MCIA finds solutions (**u**_**k**_ and **v**) sequentially. Multiple matrices **X**_**k**_ can be weighted and concatenated to a single matrix **X** = [ω_1_^1/2^**X**_**1**_ |…| ω_K_^1/2^**X**_**K**_]. Similarly, a single feature metric **Q** could be concatenated as **Q** = [**Q**_**1**_|…|**Q**_**k**_].The first order solutions of **u**_**1**_^**1**^ to **u**_**k**_^**1**^ and **v**^**1**^ are given by the first principal component of the following eigen-system:

(3) $$w\mathrm{XQ}X^{T}\mathrm{Dv}=\lambda v$$

then the normalized auxiliary axis **u**_**k**_^**1**^ are

(4) $$u_{k}^{1}=X_{K}^{T}Dv^{1}/||X_{K}^{T}Dv^{1}||{}_{Q_{k}}\left(k=1,\ldots ,K\right)$$

Where ||•|| is the norm in the **Q**_**k**_ metric. The subsequent solutions are found with residual matrices from the calculation of the first order solution with the constraint that the remaining order axes are orthogonal with the previous sets, namely:

(5) $$v^{\mathrm{jT}}Dv^{s}=0\;\mathrm{and}\;u_{k}^{\mathrm{jT}}Q_{k}u_{k}^{s}=0\;\left(1\leq j<s\right)$$

The residual matrices used by second order solution is deflated as

(6) $${X_{1}}_{\left(\mathrm{order}2\right)}=X_{1}‒X_{1}P_{k}^{1}$$

where the projection matrix **P**_**k**_^**1**^ is

(7) $$P_{k}^{1}=u_{k}^{1}{\left(u_{k}^{1}Q_{k}u_{k}^{1T}\right)}^{‒1}u_{k}^{1}Q_{k}$$

The superscript *T* and −*1* stand for matrix transposition and matrix inversion respectively. Therefore, the formula (6) removes the dimension that is spanned by vector **u**_**k**_^**1**^ (k = 1, …, K) to get a residual matrix, which is passed to the SVD to find the second order solution. These steps are repeated until the desired number of axes (principal components, dimensions) is generated. As a result, MCIA provides a simultaneous ordination of columns (measurements) and rows (features) of multiple tables within the same hyperspace, with features or measurements sharing similar trends will be closely projected. The detailed description of MCIA and the proof that these axes are maximally co-variant are given in Chessel and Hanafi [26,35].

### Datasets

We analyzed publicly available sets of data from two studies: (i) transcriptomic [8,33,36] and proteomic [11] datasets of the NCI-60 cancer cell line panel, the latter one generated in our group, and (ii) an ovarian cancer dataset generated as part of the TCGA project [37]. In each study, there are multiple datasets measuring molecules (mRNA or proteins) from the same samples (cell lines or tumors).

### NCI-60 data

The NCI-60 panel is a collection of 59 cancer cell lines of leukemia, lymphomas, melanomas and carcinomas of ovarian, renal, breast, prostate, colon, lung and central nervous system (CNS) origin. The NCI-60 transcriptome data were downloaded from Cellminer [38] and were obtained on four different platforms; Affymetrix HG-U133 plus 2.0, HG-U133, HG-U95 and Agilent GE 4x44K [39]. Affymetrix data were normalized using GC robust multichip averaging GCRMA; [39] and Agilent data were log transformed as obtained from the Cellminer. Although data filtering is not required to perform MCIA, to facilitate data interpretation, microarray data were filtered to exclude probes that do not map to an official HUGO gene symbol. The probe with highest average value was retained when multiple probes mapped to the same gene. Filtering produced datasets of 11,051; 8,803; 9,044 and 10,382 genes on Agilent, HG-U95, HG-U133 and HG-U133 plus 2.0 platforms respectively. The lung cancer cell line NCI-H23 was excluded since its expression profile was not available on the HG-U133 platform. A Venn diagram representing the overlapping genes in the processed data for each platform is provided in Additional file 1: Figure S1.

The proteome profiles of cell lines were produced from a conventional GeLC-MS/MS approach and label-free quantification, as described in [11]. The international protein index (IPI) identifiers were mapped to official gene symbol to facilitate subsequent pathway interpretation. Data were log transformed (base 10) and no filtering or additional normalization were performed. This dataset represents 7,150 protein expressions across 58 cell line in NCI-60 panel.

### Ovarian cancer datasets

Gene expression of tumors from ovarian cancer patients were profiled using two microarray platforms (Agilent customized platform G4502A and Affymetrix GeneChip HG U133 plus 2.0) and RNA-sequencing on Illumina HiSeq platform. Data were downloaded from the NCI-TCGA data portal 07/08/2013; [370]. Patient samples (266 out of 489) that were present in all four datasets were included in the analysis. The Agilent and Affymetrix data were normalized and summarized by lowess and robust multichip averaging (RMA), respectively [40]. The transcript expression levels of the Illumina RNA-sequencing data were determined using two different pre-processing pipelines (RPKM and RSEM) denoted as RNASeq and RNASeqV2, respectively. Normalization and quantification of RNASeq followed the RPKM method [41] whereas the alignment and gene expression quantification in RNAseqV2 were obtained by MapSplice and RSEM [42,43]. In RNASeq and RNASeqV2; 20,657 and 20,135 genes were detected (before filtering). These data were filtered to exclude genes with more than 15 missing values. Only genes mapped to an official gene symbol were retained. For the features mapped to the same gene symbol, the one with the largest average expression value was kept. Remaining missing values were replaced with a positive value far smaller than the lowest expression value in each dataset (10^−15^ in RNASeq and 10^−10^ in RNASeqV2) and then, the expression values were log transformed (base 10). After filtering, the Agilent, Affymetrix, RNASeq and RNASeqV2 datasets contained 17,814; 12,042; 16,769 and 15,840 gene expression measurements respectively. The Venn diagram representing the overlap of genes in these datasets is shown in Additional file 1: Figure S2.

## Results and discussion

### Integrated analysis of the NCI-60 cell line transcriptome and proteome

The NCI-60 panel, a collection of 59 cancer cell lines derived from nine different tissues (brain, blood and bone marrow, breast, colon, kidney, lung, ovary, prostate and skin) has been extensively used in *in vitro* high-throughput drug screen assays. They have been molecularly profiled using comparative genomic hybridization array [44], karyotype analysis [45], DNA mutational analysis [46,47], transcripts expression array [33,48], microarrays for microRNA expression [8] and protein expression [11].

**Figure 1:**
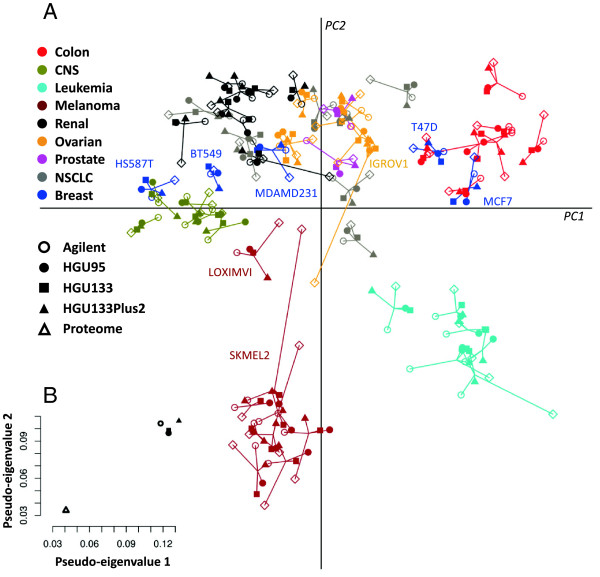
**MCIA projection plot. (A)** The first two axes of MCIA represent transcriptomic and proteomic datasets of the NCI-60 panel. Different shapes represent the respective platforms and are connected by lines where the length of the line is proportional to the divergence between the data from a same cell line. Lines are joined by a common point, representing the reference structure which maximizes covariance derived from the MCIA synthetic analysis. Colors represent the nine NCI-60 cell lines from different tissues. The epithelial and mesenchymal features are separated along the first axis (PC1, horizontal). Melanoma and leukemia cell lines were projected on the negative side of second axis (PC2, vertical). **(B)** Summarizing the concordance between platforms by representing pseudo-eigenvalue space of NCI-60 datasets. The pseudo-eigenvalue space represents overall co-structure between datasets and shows which platform contributes more to the total variance.

MCIA was applied as an exploratory analysis of four transcriptomic studies (Agilent n = 11,051; HGU95 n = 8,803; HGU133 n = 9,044 and HGU133 plus 2.0 n = 10,382) and one proteomic study (GeLC-MS/MS; n = 7,150) of the 58 cell lines. Figure 1A shows the projection of cell lines onto the first two principal components (PCs) of MCIA. Similar to the visualization employed in CIA [26], the datasets are transformed into the same projection. The coordinates of the five measurements for each cell line are connected by lines. The length of which indicates the divergence (the shorter the line, the higher the level of concordance) between the mRNAs and protein expression levels for a particular cell line. The MCIA plot of the first two principal components shows similar trends in transcriptome and proteome profiles, indicating that the most variant sources of biological information were similar. Cell lines originating from the same or closely related anatomical source of tissue were projected close to each other and converged into groups. The colon, leukemia, melanoma, CNS, renal and ovarian cell lines segregated largely according to their tissue of origin. Seven out of eight melanoma lines clustered together, and the remaining one, LOX-IMVI, has been reported to lack melanin production [49]. These results are consistent with independently performed hierarchical clustering analysis (Additional file 1: Figure S3).

There was greater divergence in the cell lines from tumors with more intrinsic molecular heterogeneity (e.g. breast and NSCLC cell lines). The transcriptome and proteome profiles of the individual breast and lung cell lines were projected close in space demonstrating that the expression profiles shared a high degree of consensus. The tight projection of multiple data types from diverse technology platforms provides evidence that the observed spread of cell lines reflected the biological variance (tumor cell lines heterogeneity), as opposed to inter-study technical or other stochastic variance. For instance, we observed that the estrogen receptor positive breast cancer cell line MCF7 displays an epithelial phenotype and clustered to colon cancer lines. In contrast, the cell line negative for the estrogen receptor, HS578T, clustered with the stromal/mesenchymal cluster of glioblastoma and renal tumor cell lines. This suggests that HS578T exhibits more invasive mesenchymal features compared to T47D and MCF7.

### Overall co-structure comparison using MCIA

Each PC has an associated eigenvalue which represents the amount of variability contained in that PC. The first three PCs of the MCIA accounted for 17.4%, 14.2% and 9.7% of variance respectively (each eigenvalue divided by the sum of all eigenvalues; Additional file 1: Figure S4). The observation that the first two PCs capture less than a third of the structure in the datasets (Figure 1A) reflects the complexity inherent in cell lines of 58 tumors from nine different organs. In order to identify the contribution of each dataset to the total variance, that is, to what extent each dataset deviates or agrees with what the majority of datasets support, we extracted the MCIA pseudo-eigenvalues. Figure 1B shows the pseudo-eigenvalues associated with the first two principal components of each dataset. Comparison within microarray data revealed that Affymetrix HGU133 Plus 2.0 accounts for the highest variance on axis 1 and 2, possibly because this platform contains informative features, or features that are poorly detected or absent on other platforms. We observed that the similarity within transcriptome datasets is greater than the similarity between transcriptome and proteome data, which is consistent with the results shown by Figure 1A.

One of the most attractive features of MCIA is that it can be used to highlight lack or presence of co-structure between datasets, thus it allows selection of the strongest features from each dataset for subsequent analysis. For instance, we observed in particular, large variation between the protein and transcript expression patterns of two cell lines, melanoma SKMEL2 and ovarian IGROV1. The proteome coordinates of SKMEL2 were close to the origin and far from the transcriptomic data that was projected on the negative end of PC2 with the other melanoma cell line data. The poor information content in proteome data of the SKMEL2 cell line could reflect the lack of expression of melanin related genes on protein level. Similarly, the incongruence of the proteome and transcriptome data of the ovarian cell line IGROV1 may be due to expression of less epithelial markers that projected on the positive direction of axis 2.

To characterize the overall correlation between each pair of high dimensional data we calculated the pair-wise RV coefficient, a multivariate generalization of the squared Pearson correlation coefficient [50]. For each pair of datasets, the RV-coefficient is calculated as the total co-inertia (sum of eigenvalues of co-inertia, i.e. sum of eigenvalues of the product of two cross product matrices) divided by the square root of the product of the squared total inertia (sum of the eigenvalues) from the individual analysis. As the co-structure between two datasets increases, the RV score move towards to 1. A zero RV score indicates no similarity. The overall similarity in structure between microarray data was higher than the similarity between microarray and proteomics data; average RV coefficient > 0.9 and 0.76 respectively (Additional file 1: Figure S5).

When MCIA was performed on the same transcriptome data and the subset of proteome data that were quantified in all 58 cell lines (n = 524 proteins, no missing values), the filtered proteome data had a higher consensus to the co-structure and increased pseudo-eigenvalues (Additional file 1: Figure S6).

### MCIA axes describe biological properties

**Figure 2:**
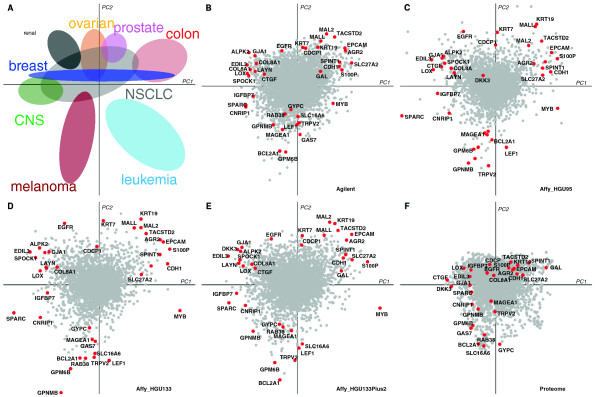
**Detecting robust markers defining major trends using MCIA. (A)** Shows the projection of the respective cell lines from the NCI-60. Colors represent tissue types as in Figure 1. **(B-E)** represent the coordinates of genes in transcriptomic data and **(F)** shows proteins from proteomics dataset. The top genes/proteins at the end of each MCIA axes are labeled in red, indicating that those features were presented in at least four platforms and located at the same direction from the origin.

In contrast to traditional clustering methods, MCIA projects the original data onto a lower dimensional space, maximizing the covariance of each dataset with respect to the reference structure. In MCIA plots, a gene that is highly expressed in a certain cell line will be projected in the direction of this cell line and the greater the distance from the origin, the stronger the association. In order to identify biomarkers that are highly associated with cancer cell lines of different origins, we examined the feature space of mRNAs and proteins that were projected in the same direction and space (Figure 2).

The first axis (PC1, horizontal axis), which explains the largest variance, separated cells with epithelial or mesenchymal characteristics, suggesting that epithelial-mesenchymal transition (EMT) is an essential mechanism defining different classes of cancers (Figure 2A). EMT has been shown to play an important role in epithelial cell malignancy and metastasis [51]. Epithelial markers, including SLC27A2, CDH1, SPINT1, S100P and EPCAM had high weights on the positive side of PC1 (Figures 2B-F). At the opposite end, mesenchymal and collagen markers, including GJA1, which is involved in epicardial to mesenchymal cell transition, and TGFβ2 were observed (Additional file 2: Table S1). The second (vertical) axis, PC2, clearly separated melanoma and leukemia from other epithelial cancer types. The strongest determinant of the vertical axis is a set of melanoma-related genes, namely melanoma-associated antigens (MAGEA), melanogenic enzyme (GPNMB) as well as TYR, DCT, TYRP1, MALANA, S100B and BCL2A1. The top 100 genes with greatest weights on PC1 and PC2 were selected from each dataset (Figures 2B-F) and the full list of markers is provided in Additional file 2: Table S1. Among 1,377 selected genes, 145 were measured in three or more datasets. MCIA enables the study of the union of features from all studies. Among the NCI-60 datasets, less than 12% of the total 17,805 genes studied were measured in all five datasets. By observing highly ranked genes across studies, one can identify robust markers that could be subject to further analysis.

### Integration of proteomics and transcriptomics complements the biological information

**Figure 3:**
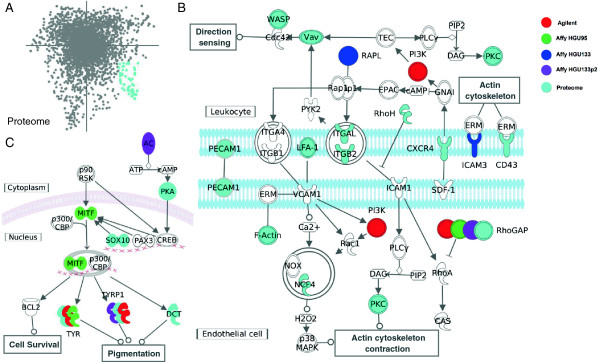
**Integrative pathway analysis. (A)** Shows the coordinates of proteins from the proteomics dataset (see as Figure 2F), where proteins from the leukemia tail are highlighted. In contrast to the microarray data, leukemia features are clearly represented in the proteome dataset. **(B)** The leukocyte extravasation signaling pathway is significantly enriched in analysis resulting from integration of leukemia features from all platforms. Colors indicate features from different datasets. **(C)** Melanocyte development and pigmentation signaling pathway was enriched in melanoma genes.

To further evaluate the biological significance of the features selected by MCIA, we employed Ingenuity Pathway Analysis (IPA: http://www.ingenuity.com) to discover significant canonical pathways which discriminate different cell lines (Figure 3). In MCIA plots, samples and features are projected onto the same space. The genes with strongest association to a cell line are those projected in the same direction and have the highest weights (greater distance from the origin). As features have been transformed on the same scale, the union of features from each individual dataset can be easily extracted and concatenated to provide greater coverage in pathway analysis. Features strongly associated to each tissue type from both transcriptome and proteome datasets can be concatenated and mapped to signaling pathways. There is no requirement to extract equal numbers of features from each data type. For example we observed that features strongly associated with leukemia related features tended to be enriched in the proteins (Figure 3A). The most extreme features associated with the leukemia cell lines were selected from all platforms using their coordinates and were subjected to the functional and pathway analysis. The full list of features, the coordinate feature selection criteria and their functional and pathway analysis are provided in Additional file 3: Tables S2 and Additional file 4: Table S3.

Complementary information can be obtained by integrating data from different platforms and data types which increases the genome coverage and power of subsequent pathway analysis. While numerous genes were over-expressed in both the transcriptome and proteome data, some (HCLS1, PECAM and two integrins, ITGAL, ITGB2) were identified exclusively in the proteome dataset (Figure 3A). We observed that leukocyte related biological functions, such as activation of mononuclear leukocyte, mobilization of Ca^2+^ and activation of lymphocyte were most strongly associated with the leukemia cell lines (Additional file 3: Table S2). Enrichment analysis suggested that the most significantly enriched pathways are, leukemia extravasation signaling pathway (*p* = 1.04^−11^; Figure 3B), which is responsible for leukocyte migration and related to metastasis in leukemia cell lines [52], T cell receptor signaling (*p* = 5.25^−5^) and iCos-iCosL signaling in T helper cells (*p* = 8.32^−5^; Additional file 4: Table S3).

To further demonstrate the advantage of combining multiple layers of information in pathway analysis, we performed identical analysis only based on transcriptome markers from all of the four microarray studies. Although leukocyte extravasation signaling was still the most enriched pathway, it did not reach the same level of significance (p = 1.14^−4^). In addition, pathways that are not strongly associated with leukemia were also significantly enriched (*p* < 0.01; hereditary breast cancer signaling and NFAT in Cardiac Hypertrophy). Several pathways that are associated with leukemia and were detected in the combined analysis were absent, including NF-kB pathway and PI3K Signaling in B lymphocytes (Additional file 4: Table S3).

We repeated this analysis on the set of MCIA discovered features associated with melanoma (Additional file 3: Table S2). The selected features comprised of proteins and genes that are highly expressed in melanoma cell lines, such as TYR, TYRP1 and BCL2A1. These were significantly enriched in the biological functions or pathways associated with eumelanin biosynthesis and disorder of pigmentation including the melanocyte development and pigmentation signaling pathway (Additional file 3: Table S2; Figure 3C). Melanocytic development and pigmentation is regulated in large part by the bHLH-Lz microphthalmia-associated transcription factor (MITF) and MITF activity is controlled by at least two pathways: MSH and Kit signaling. BCL2A1 is transcriptionally activated by MITF and serves as an anti-apoptosis factor [53]. Interestingly, the upstream regulator of MITF, lEF1, was also consistently identified on the same direction in all transcriptome datasets (Figure 2). It is of note that, although all five datasets contributed to the coverage of this pathway, MITF was solely detected in the Affymetrix data. MCIA can increase coverage and, the power of pathway (and other annotation) analyses as it does not require mapping or pre-filtering of features to the subset common to all datasets. MCIA allows easy integration of multiple omics levels to identify classes that are relevant in the given biological context.

### Comparison of MCIA and regularized generalized canonical correlation analysis (RCGGA)

In generalized canonical correlation analysis (GCCA) several sets of variables are analyzed simultaneously. Several generalizations of CCA have been described. These employ different methods, including sum of correlations (SUMCOR), sum of squared correlations (SSQCOR) and sum of absolute value correlations (SABSCOR) [29]. Recently Tenenhaus and coworkers introduced regularized generalized canonical correlation analysis (RGCCA) to generalize RCCA to multi-block data analysis of data where the number of variables exceed the number of cases [29]. We compare MCIA to several RGCCA methods that are defined by different shrinkage parameters and optimization criteria (Additional file 1: Figure S7-S9).

First, we compared three different optimization criteria in RGCCA, namely SUMCOR, SABSCOR, SSQCOR with MCIA. As depicted in Additional file 1: Figure S7, the SUMCOR method and MCIA algorithm consistently return similar results with positively correlated axes (Additional file 1: Figure S7). Also the identified components from the SABSCOR and SSQCOR methods are always highly correlated to the MCIA results, but it is important to note that the correlation could be either positive or negative. This is inconvenient for the comparison and integration of multiple omics datasets, as the components from one dataset might be inverted in another dataset.

By tuning the shrinkage parameter т, which can range from 0 to 1, RGCCA balances optimizing the intra-table and inter-table covariance. Additional file 1: Figure S8 and S9 show that the identified components are nearly identical across datasets for т = 0. The smaller the shrinkage parameter т, the higher is the correlation between neighboring components from different datasets. But the variance of each individual dataset is less well explained by the components. In contrast, the results of RGCCA with a shrinkage parameter of т = 1 are very similar to MCIA results. In this case, RGCCA gives priority to finding a component that explains its own block well [29]. Similarly, MCIA maximizes the variance within each table and the covariance of components of each table with a consensus reference structure through a synthetic analysis. It is important to note that in omics data analyses, the number of features is generally much larger than the number of observations. Therefore, a low т should be avoided as it results in overfitting of the data and apparently perfect correlations, which rarely represent meaningful information.

### Integrated analysis of microarray and RNA-sequencing ovarian cancer datasets

**Figure 4:**
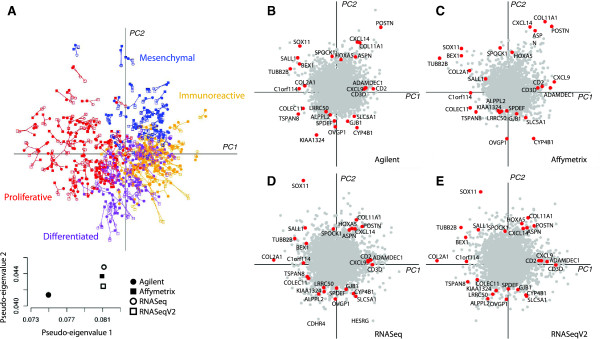
**Cross-platform comparison of transcriptional expression profiles of ovarian cancer using MCIA. (A)** Visualization of the concordance of patient gene expression profiles from multiple platforms. The samples are colored according to four subtypes of patients [56]. Axis 1 clearly separates the proliferative and immunoreactive subtypes, whereas the mesenchymal and differentiated subtypes are separated by axis 2. The inset represents the pseudo-eigenvalues of each dataset on the first two PCs. **(B-E)** Show the coordinates of genes from each platform. Top consensus genes, common to all platforms from the end of each axis, are colored and labeled.

In the ovarian cancer datasets, MCIA was applied to several microarray and RNA-seq gene expression datasets; Agilent, Affymetrix, RNASeq, RNASeqV2 which contained 17,814, 12,042, 16,769, and 15,840 genes respectively. In the MCIA space, the first PC (horizontal axis) accounted for 19.6% of the total variance and the second PC (vertical axis) accounted for 10.6% of variance (Additional file 1: Figure S10). In comparison to microarray data, RNA sequencing data typically contains many missing values. These are generated when multiple experiments are combined. We excluded genes (rows) with high number of missing values. After filtering genes with more than 15 missing values in RNA-seq data, the four datasets contributed similarly to the total variance (Figure 4 and Additional file 1: Figure S11). Among the two RNA-seq datasets, RNASeq consistently tended to be more variant than RNASeqV2 on PC1-5 (Additional file 1: Figure S12). RNASeq and RNASeqV2 were generated from the same Illumina RNA-sequencing data but using two different pre-processing approaches. MCIA results indicated that normalization and quantification with the RPKM method (RNASeq) outperforms MapSplice and RSEM (RNASeqV2). The informativeness or variance in RNA sequencing data tended to be sensitive to pre-processing and filtering algorithms which is expected given that methods for processing these data are still emerging. In addition, Affymetrix profiles were generally more variant than Agilent as indicated by greater pseudo-eigenvalues on PC1-3. When the microarray and RNASeq data were compared, we detected several outlier genes that were highly variant on PC1 and PC2 on RNASeq but absent on the microarray platforms. These include CDHR4 and HESRG which are highly expressed by the differentiated subtype (Figure 4) [54].

### MCIA identified ovarian subtypes

We applied MCIA to compare the consistency and discrepancy in gene expression profiles of ovarian cancer tumors obtained by RNA-sequencing and Affymetrix and Agilent microarray technologies (Figure 4A). The results revealed high overall similarity in structure between the four datasets and three platforms.

Recent microarray gene expression profiling studies have reported four subtypes of ovarian cancer (proliferative, immunoreactive, mesenchymal and differentiated) [37,55]. These HGS-OvCa subtypes can be clearly distinguished along the first two MCIA axes (Figure 4A). The first axis generally separated samples with immunoreactive versus proliferative characteristics. Whereas the second axis distinguished tumors with a mesenchymal subtype which show a short survival time [56] from the differentiated ovarian cancer samples. Consistent with other studies, MCIA identified large overlap between the four subtypes, indicating that most samples exhibited features of multiple subtype signatures [56]. In order to find whether this classification correlates with clinical factors, we compared the first two PCs with clinical records provided from the TCGA data portal and the Verhaak study [56]. This comparison revealed that age at diagnosis was significantly negatively correlated with PC1 and positively correlated PC2 (Pearson correlation *p* = 1.29^−3^ and *p* = 3.56^−4^ respectively), suggesting that differentiated and immunoreactive patients tend to present at younger age. The percentage of stromal cells is positively correlated with PC2 (Pearson correlation *p* = 1.79^−3^), which is in consensus with the mesenchymal subtype having greater percentage of stromal cells [56]. Other clinical factors, such as somatic mutation, drug treatment and tumor stages did not significantly correlate with either axis.

### MCIA suggests robust subtype biomarkers

Both microarray and RNA sequencing data resulted in a similar ordination of tumor samples in the MCIA space. In order to identify which genes contribute significantly to the divergence of samples, we examined the gene expression variables superimposed onto the same space (Figure 4B-E). The top 100 genes from the end of each axis were selected. The full list of selected genes and their enriched pathways are provided in Additional file 5: Table S4. Each dataset contained different genes. Approximately 47% of genes (9,755 genes) were measured on all four datasets (Additional file 1: Figure S2). Among 1096 genes selected as the top 100 genes from each datasets on PC1 and PC2 only 82 genes were in at least three platforms and 27 (2.5%) were present in all datasets. Several of these “robust” markers, have been previously implicated in ovarian cancer [37,56]. Many T-cell activation and trafficking genes, such as CXCL9, CD2 and CD3D were projected onto the positive end of the first axes, which represented the immunoreactive subtype tumors. MCIA revealed new markers that might be associated with the immune system, including SH2D1A, RHOH, SAA1, SAA2 and GNLY. This is further corroborated by numerous GO terms significantly associated with genes on this end of the axis (DAVID functional annotation) [57]. For instance, significantly enriched gene sets include glycoprotein, chemotaxis, defense and immune response (FDR < 0.01, Additional file 5: Table S4). The genes at the opposite end of the MCIA axes included transcriptional factors SOX11, HMGA2, along with several cell cycle promoters, such as BEX1, MAPK4 as well as nerve system development regulators (TBX1, TUBB2B), which characterize the proliferative subtype. Genes that are expressed on the positive end of axis 2, such as POSTN, CXCL14 and HOXA5, define the mesenchymal cluster. Other potential mesenchymal subtype markers for ovarian cancer include ASPN, homeobox alpha genes as well as collagens*.* ASPN is a critical regulator of TGF-beta pathway that induces the epithelial mesenchymal transition. Gene set analysis revealed that mesenchymal genes are enriched in GO terms including cell adhesion, skeletal system development, collagen and ECM receptor interaction pathway (Additional file 5: Table S4).

The robust markers at the differentiated end include oviductal glycoprotein 1 (OVGP1/MUC9), SPDEF, KIAA1324, GJB1 and ALPPL2, some of which have already been reported as ovarian biomarkers. For instance, OVGP1 has been suggested as a possible serum marker for the detection of low grade ovarian cancer [58]. Although the TCGA dataset is all high grade serous ovarian cancer, in our analysis, it was highly expressed in differentiated subtype. Human SPDEF protein plays a significant role in tumorigenesis in multiple cancers, including ovarian cancer and has been reported to suppress prostate tumor metastasis. A recent study on prostate cancer demonstrated that SPDEF suppresses cancer metastasis through down-regulation of matrix metalloproteinase 9 and 13 (MMP9, MMP13), which are required for the invasive phenotype of cells [59]. Our analysis implied that SPDEF and matrix metalloproteinase plays a similar role in the development of ovarian cancer. In addition, it has been shown that, in a mouse model, POSTN down-regulates ALPP mRNA [60]. POSTN and ALPPL2 were projected onto the diametral ends of axes 2, which implies that the same mechanism of regulation exists in ovarian cancer and can be exploited to distinguish subtypes. Interestingly, the DAVID gene set analysis of markers for the differentiated phenotype did not reveal as strong gene set enrichments as described for the other subtypes (lowest FDR = 0.0022 vs. 10^−47^ to 10^−9^; Additional file 5: Table S4) indicating that this subtype exhibits considerably higher degree of heterogeneity.

## Conclusion

In the present study, we described multiple co-inertia analysis (MCIA), an exploratory data analysis method that can identify co-relationships between multiple high dimensional datasets. MCIA projects multiple sets of features onto the same dimensional space and provides a simple graphical representation for the efficient identification of concordance between datasets. The sets of features may have none or few features in common. By transforming multiple sources of data onto the same scale, the most variant features are transformed onto the same scale. This allows one to extract and easily combine sets of omic features (genes, proteins, etc.) for greater power in subsequent pathway analysis. MCIA provides a consensus reference structure of datasets, revealing similar trends among multiple tables. Compared to RGCCA, we found that MCIA is most similar to the SUMCOR version of RGCCA with т = 1 in practice.

Our integrative analysis of NCI-60 cell line panel indicated that, although both transcriptome and proteome cell lines were clustered according to their lineage, they provides complementary information. We demonstrated that integrated analysis of gene and protein expression data increases the power of pathway analysis and yields more information than an analysis of gene expression alone. MCIA highly ranked the leukemia extravasation signaling pathway. This pathway was overrepresented with features that were predominantly from the proteomics data and were enriched in biological functions of “activation of mononuclear leukocyte and lymphocyte”. MCIA of high grade serous ovarian cancer revealed four previously described subtypes of ovarian cancer and provided novel subtype markers. An advantage of MCIA is that it couples multiple set of features measured on the same set of samples. Since it does not rely on feature annotation, it is not limited by the immaturity of annotations. There is no prerequisite to filter or map features (genes) to a common set thereby considerably increasing genome coverage.

In a study that compares CIA with other sparse multiple table analysis methods (sPLS and CCA-EN), LeCao *et al.* suggested that CIA may result in redundancy when it is used for feature selection since it does not include a built-in procedure for variable selection [27]. Similarly, MCIA does not impose any sparsity in the result, so MCIA selects much more features than methods introducing the Lasso penalty, such as SGCCA [30] or PCCA [31]. Hence, the interpretation of MCIA selected features would have to be coupled with other methods, such as enrichment analysis, in order to reveal functional insights. We also note that the MCIA algorithm finds solutions in a sequential manner and each order of components requires a singular value decomposition (SVD) for a large dataset. The computationally intensity of the algorithm increases with sample size as more components are retained. For instance, the CPU time of analysis of the NCI-60 data with 5 kept principal components was around 68 seconds on Intel Xeon 1596 MHz using one thread of a Linux server.

In conclusion, we believe MCIA is a useful method for integration of multiple omics datasets where the same tissue or cell lines have been assayed multiple times. MCIA is available to the community via an R-Bioconductor (“omicade4”) package which includes documentation and a vignette.

### Availability of supporting data

The microarray data of NCI-60 cell lines are available through CELLMINER (http://discover.nci.nih.gov/cellminer/home.do). The NCI-60 proteomic data can be downloaded from http://wzw.tum.de/proteomics/NCI60/ as well as from https://www.proteomicsdb.org. The ovarian cancer data are available through the TCGA download portal (https://tcga-data.nci.nih.gov/tcga/tcgaHome2.jsp).

## Abbreviations

CCA: Canonical correlation analysis; CCA-EN: Canonical correlation analysis with elastic net penalty; CIA: Co-inertia analysis; CNS: Central nervous system; COA: Correspondence analysis; CPCA: Consensus principal component analysis; EMT: epithelial-mesenchymal transition; ENCODE: The encyclopedia of DNA elements; GCCA: Generalized canonical correlation analysis; gcRMA: GC robust multichip averaging; GeLC-MS/MS: In-gel digestion and liquid chromatography tandem mass spectrometry; HGS-OvCa: High grade serous ovarian cancer; ICA: Independent component analysis; IPA: Ingenuity pathways analysis; IPI: International protein index; MCIA: Multiple co-inertia analysis; MS: Mass spectrometry; NCI: The national cancer institute; NSC: Non-symmetric correspondence analysis; NSCLC: Non-small-cell lung carcinoma; PAGE: Polyacrylamide gel electrophoresis; PC: Principal component; PCA: Principal component analysis; PCCA: Penalized canonical correlation analysis; RGCCA: Regularized generalized canonical correlation analysis; RMA: Robust multichip averaging; RNASeq: RNA sequencing; RPKM: Reads per kilo base per million; RSEM: RNA-Seq by Expectation Maximization; SVD: Singular value decomposition; SGCCA: Sparse generalized canonical correlation analysis; sPLS: Sparse partial least square; TCGA: The cancer genome atlas.

## Competing interests

The authors declare that they have no competing interests.

## Authors’ contribution

CM carried out the analysis as a PhD student in the group of BK and wrote the manuscript. BK provided input regarding the interpretation of the results. AC and AMG made numerous important intellectual contributions, provided input for both the design of the study and drafting of the manuscript. AMG designed and supervised the study and wrote the manuscript. All authors read and approved the manuscript.

## Supplementary Material

Additional file 1 **Supplementary information. Figures S1–S12.** Click here for file

Additional file 2: Table S1 Full list of biological markers highly weighted on each MCIA axis of the NCI-60 data. Click here for file

Additional file 3: Table S2 Full list of Leukemia and Melanoma markers (including the corresponding selection criteria) and the IPA functional analysis. Click here for file

Additional file 4: Table S3 Pathway analysis of leukemia markers. Click here for file

Additional file 5: Table S4 Functional analysis of the ovarian subtype markers. Click here for file
