# Supplementary material for: A multivariate approach to the integration of multi-omics datasets
Source: BMC Bioinformatics. 2014 May 29;15:162. doi: 10.1186/1471-2105-15-162 (PMC4053266; doi:10.1186/1471-2105-15-162)
Supplement: Additional file 1 — Supplementary information. Figures S1–S12. [file 1471-2105-15-162-S1.docx]

Supplementary information

**A multivariate approach to the integration of multi-omics datasets**

Chen Meng^1^, Bernhard Kuster^1,2.3^, Aedín C.Culhane^4,5^ and Amin Moghaddas Gholami^1,*^

^1^ Chair of Proteomics and Bioanalytics, Technische Universität München, Freising, Germany

^2^ Center for Integrated Protein Science Munich, Germany

^3^ German Consortium for Translational Cancer Research, Germany

^4^ Department of Biostatistics and Computational Biology, Dana-Farber Cancer Institute, Boston, MA02215, USA

^5^ Department of Biostatistics, Harvard School of Public Health, Boston, MA 02215, USA

* To whom correspondence should be addressed. Tel: +49 8161 712065; Fax: +49 8161 71 5931; Email: [amin@tum.de](mailto:amin@tum.de)


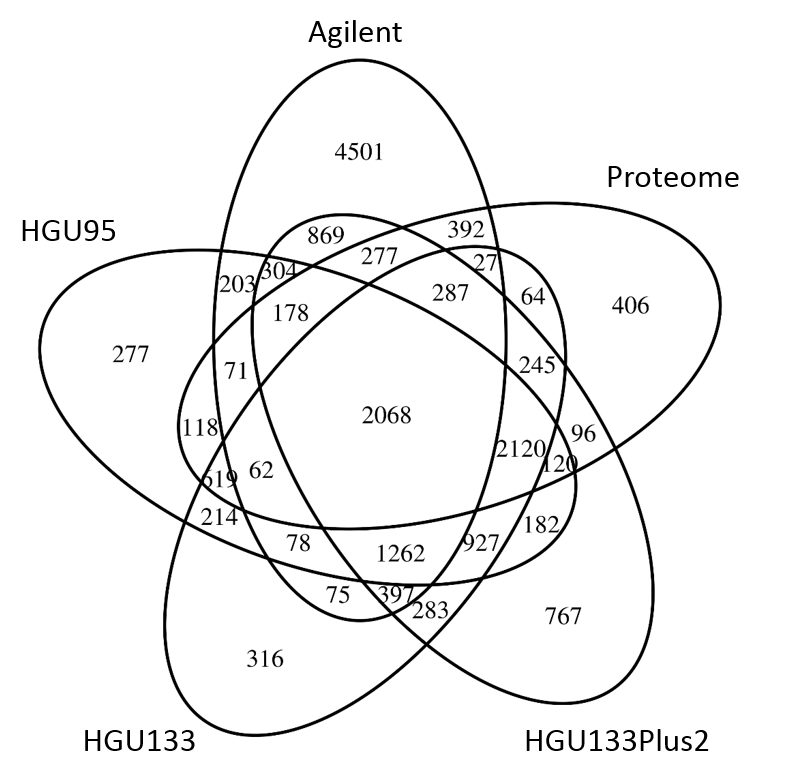


**Figure S1** Venn diagram representing the shared number of identified genes and proteins in the NCI-60 datasets (total 17,805 genes).


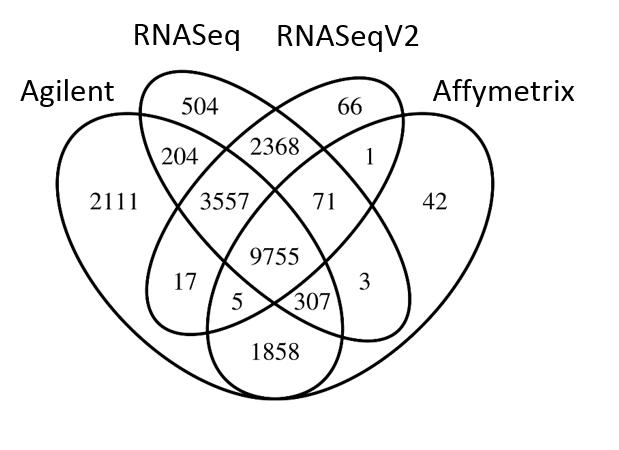


**Figure S2** Venn diagram representing the total 20,869 number of detected genes in the four ovarian datasets.


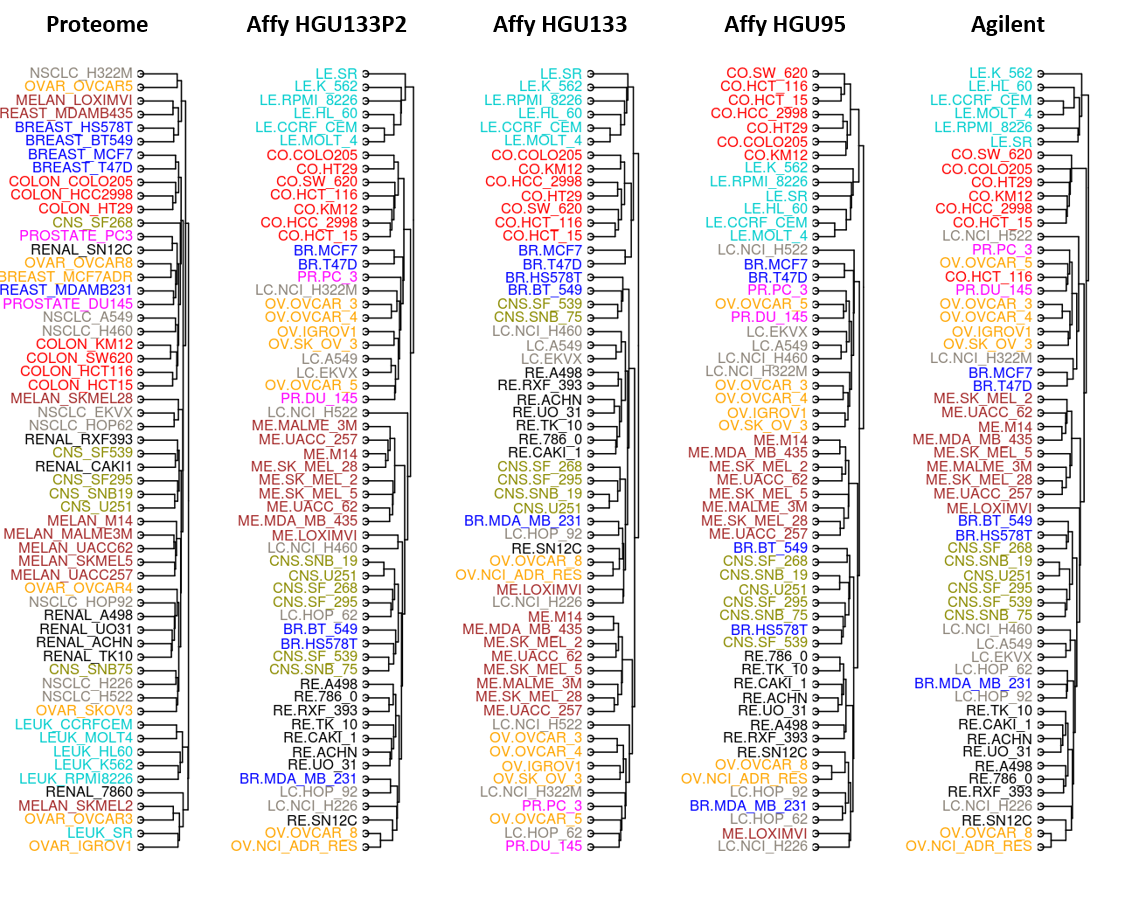


**Figure S3** Hierarchical clustering of mRNA and protein expression profiles of the NCI-60 dataset. Dendrograms showing the average linkage hierarchical clustering using Euclidean distance. The cell lines are colored as in Figure 1.


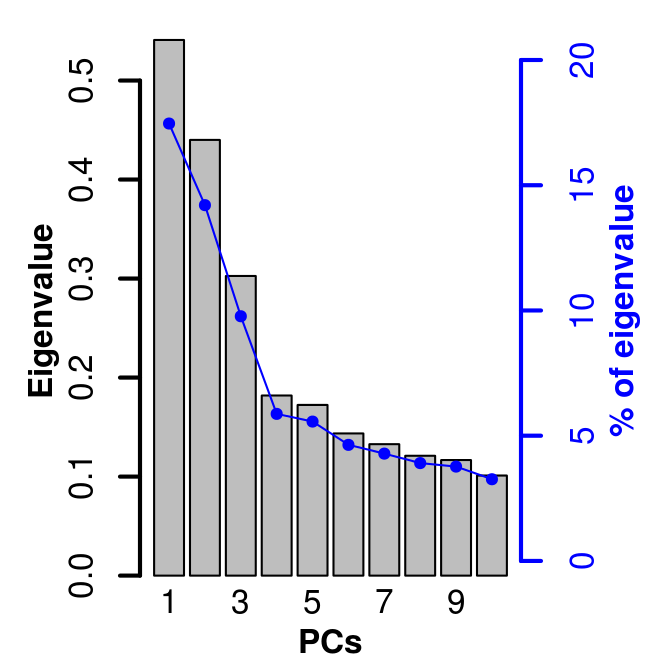


**Figure S4** Pseudo-eigenvalues of the NCI-60 data, including four microarray datasets and one proteomic data. Each pseudo-eigenvalue is associated with a principal component (linearly uncorrelated variables defined by MCIA) indicating the variance explained by each PC. Barplot shows the pseudo-eigenvalues (left axis) and blue line corresponds to the percentage of variance of each PC, calculated as the eigenvalue divided by sum of all eigenvalues. The first three PCs represent 17.6%, 14.2% and 9.7% of the total inertia.


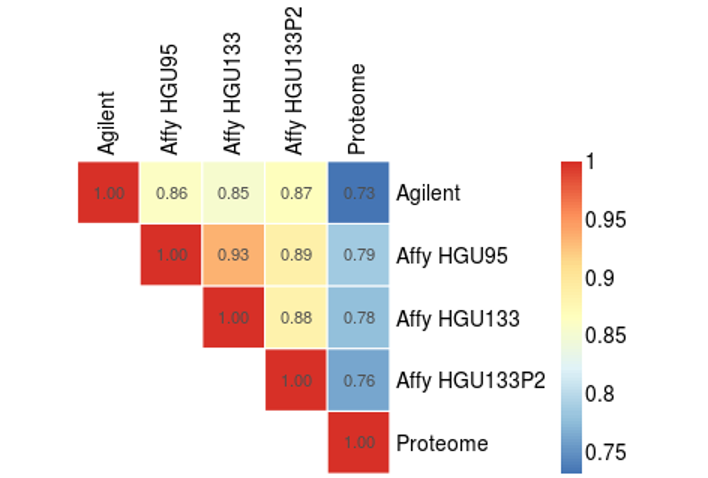


**Figure S5** Heatmap shows the RV coefficients between each pair of normalized datasets, representing a high degree of overall similarity in the structure of transcriptomics and proteomic datasets.


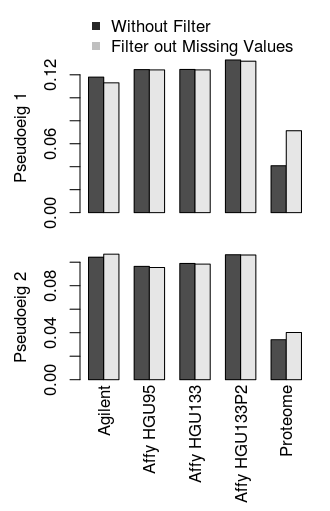


**Figure S6** Shown are the pseudo-eigenvalue space of the NCI60 data representing the contribution of each dataset to the first principal component (top) and second principal component (bottom). In both cases filtering out missing values in the proteome data increased the variance.


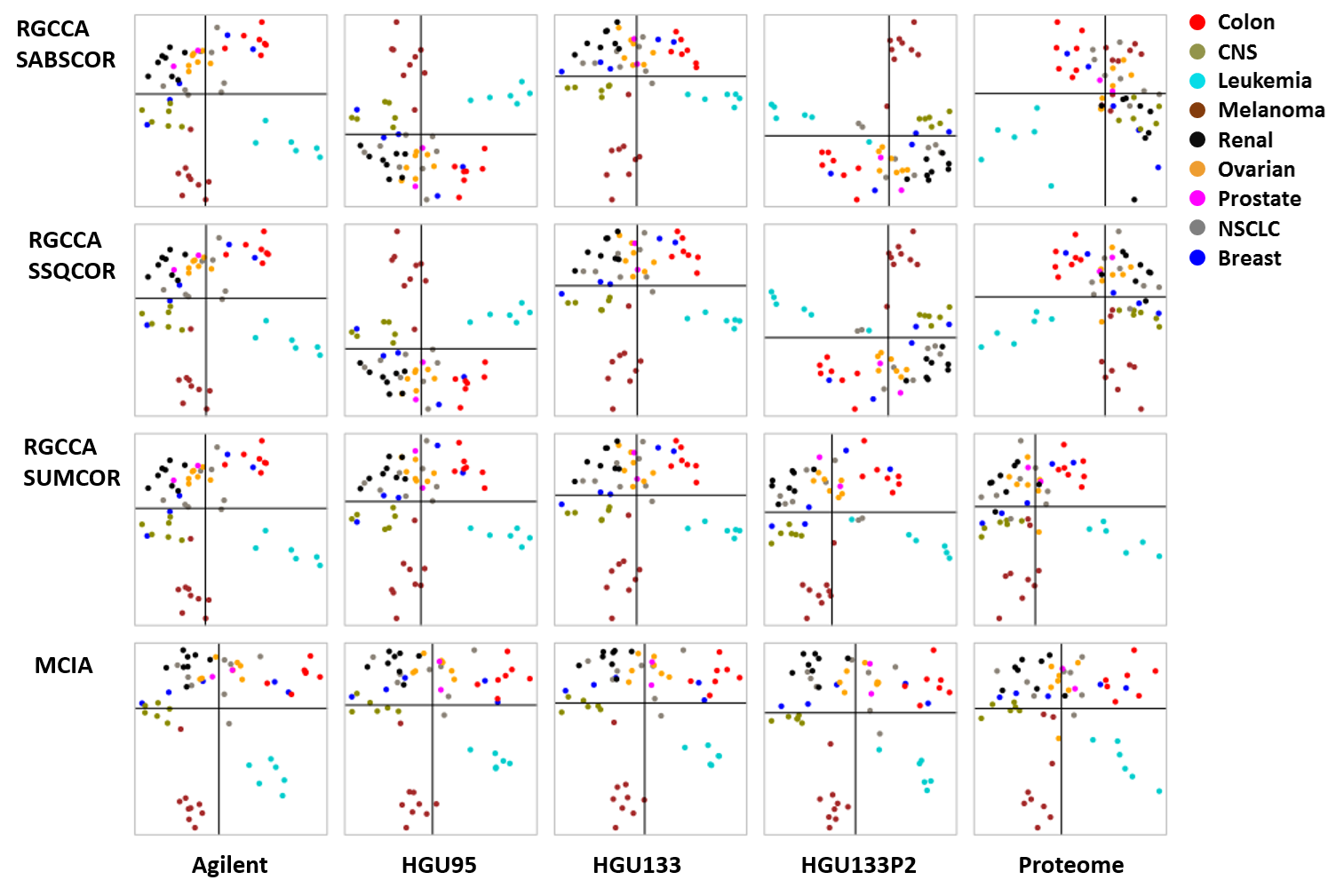


**Figure S7** Comparison of three optimization criteria for RGCCA with MCIA using the described NCI-60 datasets. The sample space of each dataset is plotted separately (columns). The following three RGCCA optimization criteria (rows) were compared: sum of absolute correlation (SABSCOR), sum of squared correlation (SSQCOR) and sum of correlation (SUMCOR). SABSCOR and SSQCOR maximize the absolute value and square of correlations between axes, therefore, axes could be either negatively or positively correlated, as shown by the inverted axes across datasets. In contrast, the SUMCOR method identifies positively correlated components, which is closest to the MCIA. In all cases of RGCCA, only complete design was considered and the shrinkage parameter was set to т = 1 in all cases (results were generated by R package “RGCCA”).


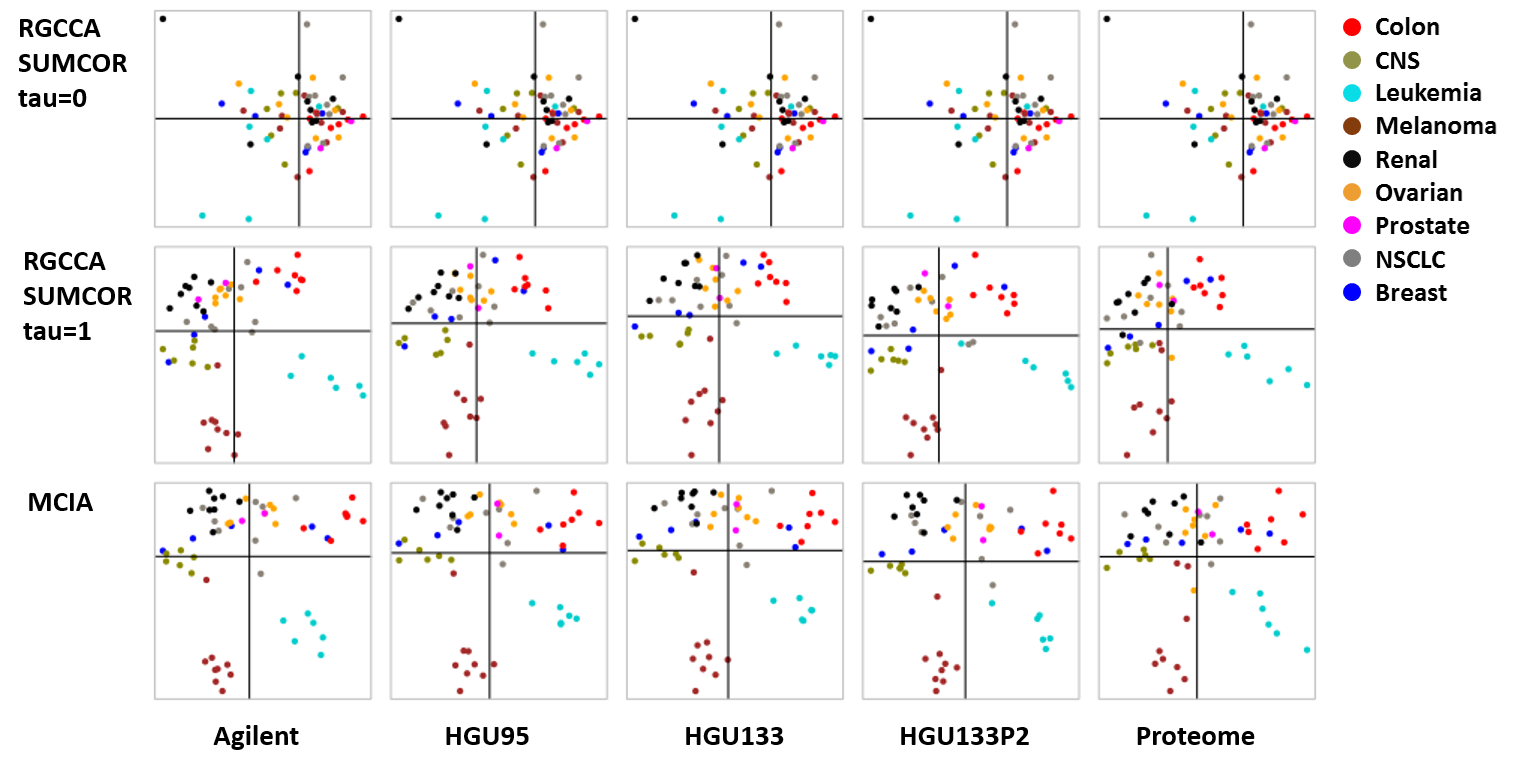


**Figure S8** Comparison of different shrinkage parameters (т) for RGCCA as well as MCIA using the NCI-60 data. The sample space of each dataset is plotted separately (columns). When т = 0, the components of all datasets are perfectly correlated. When т = 1, the result is comparable with MCIA. In all cases, complete design and the SUMCOR method were used (results were generated by R package “RGCCA”).


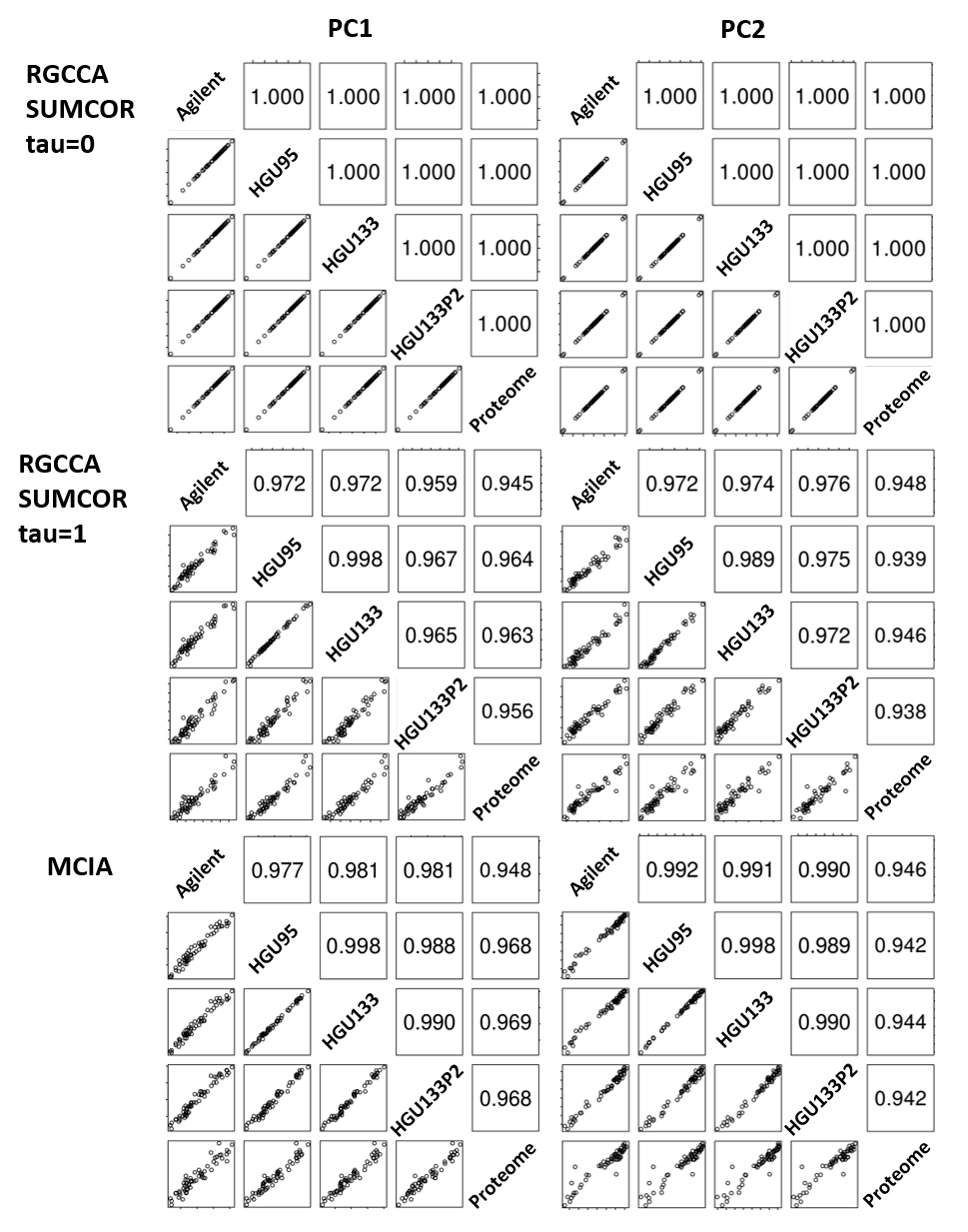


**Figure S9** The scatterplots and correlation coefficients of RGCCA and MCIA for first and second PC. The first PC (PC1, left column) and second PC (PC2, right column) across five NCI-60 datasets were generated by RGCCA with т = 0 (top row); RGCCA with т = 1 (middle row); and MCIA (bottom row). When т = 0, the correlations coefficients are always 1. If т = 1, the axes are also well correlated but the correlation coefficients are less than 1. The correlations of MCIA axes are closer to the case of RGCCA with т = 1.


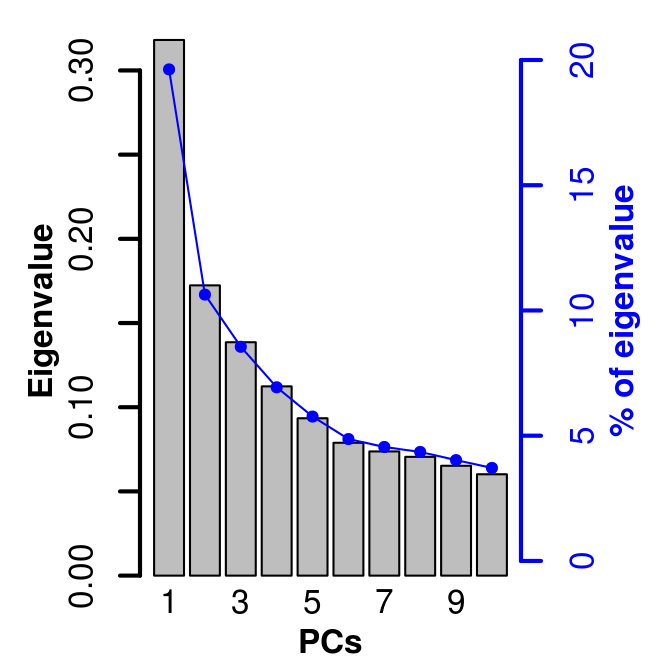


**Figure S10** The figure represents pseudo-eigenvalue of the ovarian data, including two microarray and two RNA sequencing data. Each pseudo-eigenvalue is associated with a principal component representing the explained variance by each PC. The scree plot shows the pseudo-eigenvalues (left axis); blue line indicates the percentage of variance of each PC. The first three PCs represent 19.3%, 10.63% and 8.5% of the total inertia.


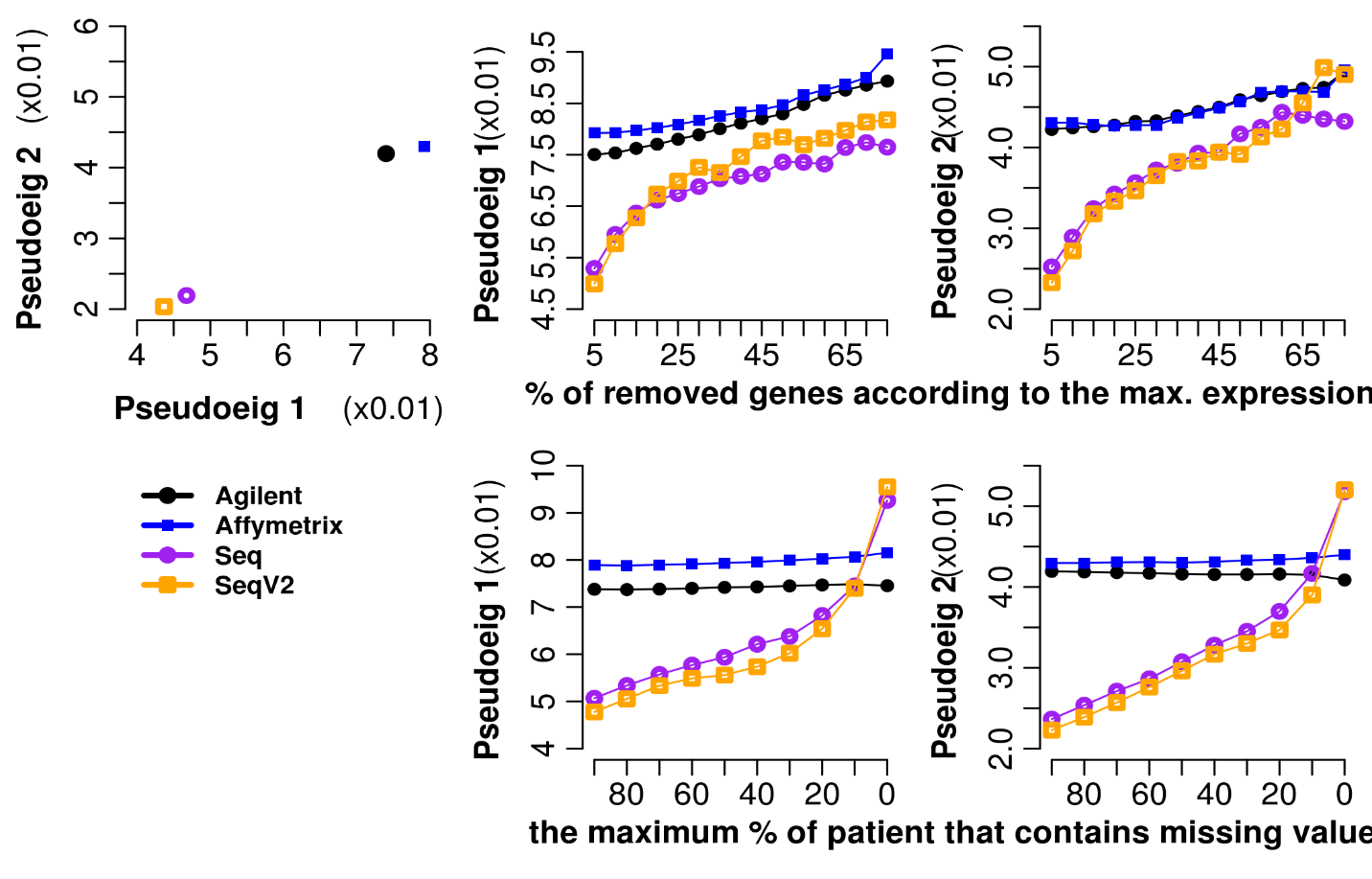

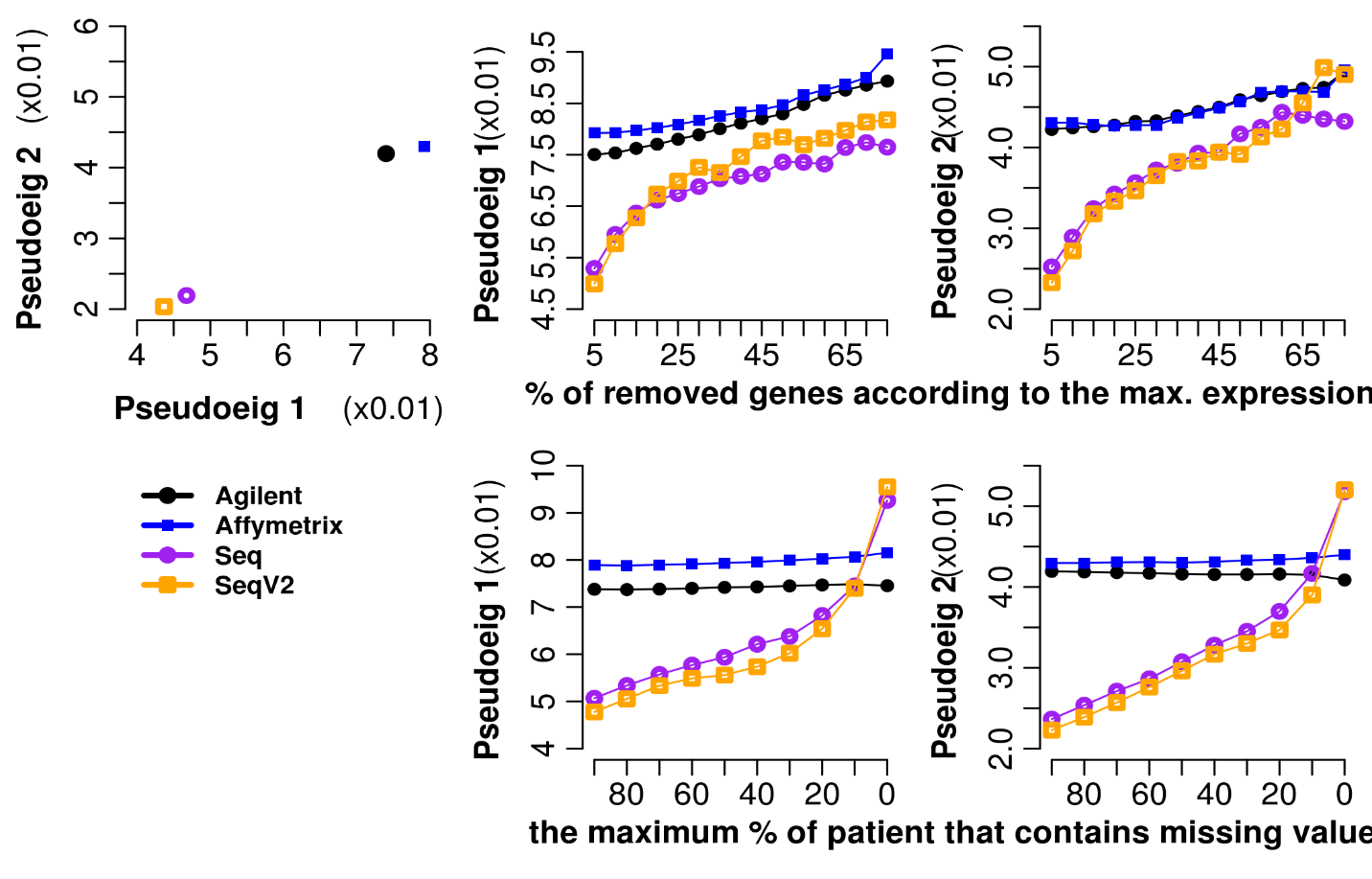


**Figure S11** The figure shows that filtering out missing values in the RNA sequencing data increases the co-structure between microarray and RNA sequencing data. In the RNA sequencing data, genes with the amount of missing data over the range of 0-90% are excluded. The eigenvalue associated with PC1 (left) and PC2 (right) are compared. Plot shows that when filtering out genes with more than 27 missing values (10%), the eigenvalues of the four platforms become more similar.


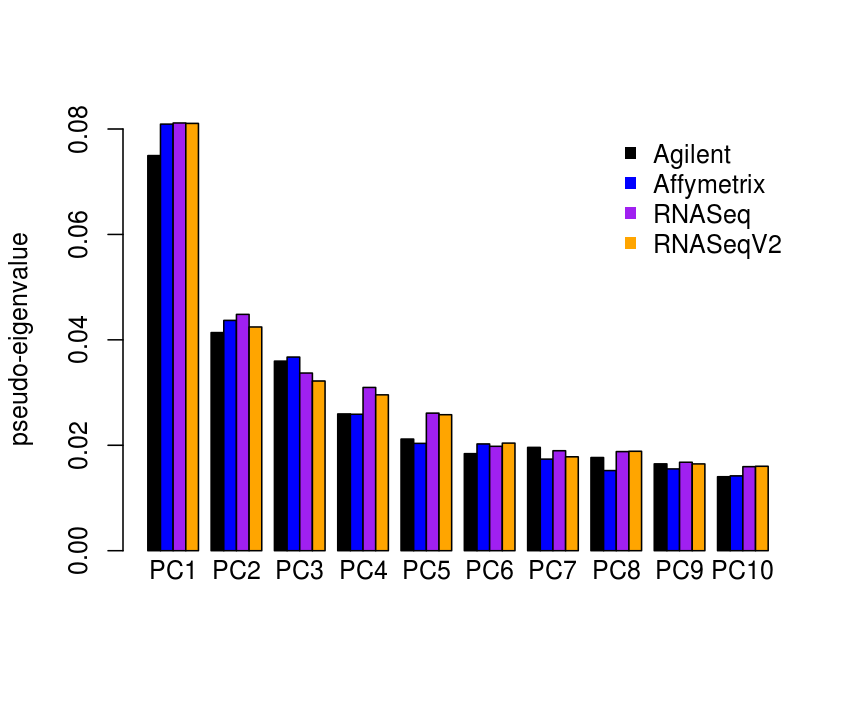


**Figure S12** Barplot representing the first 10 pseudo-eigenvalues of ovarian transcriptome on the MCIA space. The plot shows that RNASeq was slightly more informative than RNASeqV2 on PC1-5 whereas Affymetrix gene expression profile represented more variant on the first three PCs.
